# Healthcare Professionals' Views on the Management of Medication Complexities in the Elderly With Mental Health Disorders: A Cross-Sectional Study

**DOI:** 10.3389/fpsyt.2022.885216

**Published:** 2022-05-23

**Authors:** João Pedro Aguiar, João Gama Marques, Hubert G. M. Leufkens, Filipa Alves da Costa

**Affiliations:** ^1^Research Institute for Medicines (iMED.ULisboa), Faculdade de Farmácia, Universidade de Lisboa, Lisboa, Portugal; ^2^Centro de Investigação Interdisciplinar Egas Moniz (CiiEM), Instituto Universitário Egas Moniz (IUEM), Caparica, Portugal; ^3^Serviço de Psiquiatria Geral e Transcultural, Centro Hospitalar Psiquiátrico de Lisboa (CHPL), Lisboa, Portugal; ^4^Clínica Universitária de Psiquiatria e Psicologia Médica, Faculdade de Medicina, Universidade de Lisboa (FMUL), Lisboa, Portugal; ^5^Division of Pharmacoepidemiology and Clinical Pharmacology, Utrecht Institute for Pharmaceutical Sciences, Utrecht, Netherlands

**Keywords:** knowledge, potentially inappropriate medications, healthcare professionals, barriers, mental health disorders

## Abstract

**Background:**

Many challenges in elderly pharmacotherapy are identified, including the use of Potentially Inappropriate Medications (PIMs) which may increase the odds of adverse events, especially in elderly patients with mental health disorders (e. g., behavioral, and psychological symptoms of dementia–BPSD, schizophrenia, bipolar disorder). However, information on the knowledge and practice of healthcare professionals (HCPs) about this topic is still scarce.

**Methods:**

A cross-sectional study was undertaken from July-October 2019. An online questionnaire was specifically designed and validated for this study. We sought HCPs (physicians, pharmacists, and nurses) worldwide, using (a) social media, via Facebook, Twitter, and LinkedIn; and (b) email contacts of the research team (convenience sample). Either way participants were asked to share on their social media or via e-mail the questionnaires with other HCPs (snowballing sample). The survey assessed two main domains: knowledge and practice. Knowledge was evaluated by self-report (perceived knowledge by a 5-item Likert confidence scale) and using three clinical cases, scored between 0 and 30 points (each one rated from 0 to 10 points; real knowledge). Barriers in clinical practice were evaluated using a 5-item Likert scale judging practitioners' opinion.

**Results:**

A total of 165 questionnaires were collected. HCPs were mainly female (*n* = 114; 69.1%), with a mean age of 35.3 ± 11.3 years old. Seventy-two percent (*n* = 118) were pharmacists, 21.1% (*n* = 35) were physicians, and 7.3% (*n* = 12) nurses. There was a weak correlation, albeit significant, between perceived and real knowledge (r = 0.199; *p* = 0.001). The mean score of the clinical vignettes regarding elderly patients with dementia and bipolar disorder were 4.59 ± 4.08 and 4.86 ± 2.97 points, respectively. Most HCPs were classified as having an intermediate knowledge (*n* = 100; 60.6%) about medication complexities in the elderly with mental disorders. Most HCPs agreed that lack of time (81.6%; *n* = 138), lack of education and training on elderly pharmacotherapy (72.2%; *n* = 122), and lack of tools adapted to daily practice (61.8%; *n* = 105) were the main barriers.

**Conclusions:**

Most of the HCPs felt confident to manage medication complexities in elder patients with mental disorders, but only a minority obtained a good score in the knowledge assessment test. The main barriers identified included structural barriers (tools unfit for practice) and process barriers (time).

## Introduction

Population aging has been increasing worldwide in the past decades. In 2010, 524 million people were aged 65 or older – 8.0% of the world's population –, and it is estimated that by 2025 there will be a total of about 1.2 billion people aged 60 or older (1, 2). Individuals aged 80 or older are the fastest growing fraction of the population and are expected to reach 30.0% of the overall population in industrialized countries by 2050 (3, 4). Older individuals tend to present multiple chronic conditions (multimorbidity), requiring the use of multiple medications. Aging has introduced several changes in patients' physiology, which contributed to different pharmacokinetics/pharmacodynamics (PK/PD) patterns (5).

Polypharmacy can be defined based on the number of medications taken by the patient, where it is normally considered as the use of 5 or more drugs, or based on the appropriateness of the medications included, as appropriate or inappropriate polypharmacy (1, 6). Inappropriate polypharmacy is defined as the use of too many medications, including medicines where the risk of adverse drug events (ADE) outweighs the clinical benefit (7, 8). On the other hand, we can have patients that may be using potentially inappropriate medications (PIMs), i.e., medications where the risk of ADEs may outweigh the clinical benefit of its use. These medications can be classified as PIMs independently from comorbidities, or because there is a potentially inappropriate interaction with an underlying condition or another medication. Since polypharmacy includes the use of multiple drugs (normally 5 or more), at least one of them may be considered a PIM (9).

The complexity of care required by elder individuals, increase their use of healthcare services and to consult different Healthcare Professionals (HCPs). This results in the need for interprofessional collaboration among general practitioners, different specialist physicians, pharmacists, nurses, and other HCPs. However, interprofessional communication is nowadays uncoordinated and may result in an increased risk of polypharmacy and inappropriate medication use. A study conducted by Mahlknecht et al. (10) has shown that systematic education of HCPs and structured interprofessional medication review may decrease the mean number of severe drug-drug interactions as well as a decreased agitated behavior in older adults in nursing homes (10, 11).

In psychiatry, polypharmacy is normally a reality that physicians struggle to handle since many of the clinical guidelines and treatment algorithms prefer a monotherapy approach. However, in some cases, polypharmacy is clinically needed to handle persistent symptoms and nonresponse to monotherapy (12). Some studies have investigated the prevalence of polypharmacy and clinical features that may be associated with this issue and concluded that polypharmacy can be a risk in some subgroups of patients (13, 14). For example, a systematic review identified that elderly with bipolar disorder are exposed to polypharmacy, but it may be a risk depending on the type and mood episode phase of illness that the patient present. They also have shown that it depends on the type of medications used (e.g., lithium, antipsychotics) (13). Many types of polypharmacy have been identified in this area, namely, same class (e.g., the use of medication from the same class), multi-class (e.g., full dose of different medications from different classes), adjunctive (e.g., one medicine is used to treat an ADE of another), augmentation (e.g., the use of one medication at a lower dose with another at full dose) or total polypharmacy (12). Even though, it seems beneficial to use polypharmacy in patients with mental disorders (to control negative, positive, cognitive, or behavioral symptoms), there seems to be scarce evidence to support that. Some studies have shown that healthcare professionals, especially clinicians, should evaluate if polypharmacy enhances clinical outcomes or whether it promotes ADEs (12, 15).

Since some of the medications identified in several PIM-lists are used to treat several mental disorders and knowing that in older individuals they may increase the odds of ADEs, it seems to be relevant to evaluate the knowledge of healthcare professionals on the management of polypharmacy and PIMs in older patients with mental health disorders and to identify potential barriers in clinical practice.

## Materials and Methods

### Study Design

This study follows the STROBE reporting guidelines (16). A cross-sectional study was undertaken, using an online questionnaire from July to October 2019.

### Population and Sample

The target population for this study was defined as HCPs that have an active role in medication review for the elderly. Therefore, we selected physicians, pharmacists, and nurses as the main HCPs of interest. Given the difficulty in defining the theoretical sample of HCPs necessary to reach, due to limited data on the number or percentage of HCPs that have access to social media or even to the internet, we have applied a snowball sampling technique by disseminating the questionnaire via social media and via email. The database of email contacts included 17 pharmacists, 16 physicians, and 16 nurses. These participants were a mix of HCPs actively practicing at the time of study and HCPs known to have some expertise in geriatrics. We have deliberately excluded all participants that did not fit in one of those three professional categories, including students (given that they were not practicing yet), interns, and retired professionals. Our goal was to obtain at least 100 HCPs, with a balanced number between the different professions.

### Development of the Questionnaire

The self-administered online questionnaire was developed from scratch following literature review and made available bilingually (English and Portuguese) (17–19). This questionnaire collected sociodemographic characteristics and consisted of three domains: (a) perceived knowledge; (b) actual knowledge; and (c) potential barriers to PIMs' management in clinical practice. Sociodemographic variables collected included age, sex, country, professional category, practice setting, academic degree, and years of practice. The perceived knowledge domain was implemented through the presentation of statements focused on perceived facility of identifying PIMs when undertaking medication reviews, and on frequency of use of any tool to guide medication review (e.g., Beers criteria, START/STOPP criteria, PRISCUS criteria, Medscape) and then searching for agreement of HCPs. The actual knowledge domain was implemented by presenting three clinical vignettes based on real-life clinical cases where Beers criteria 2019 version had been applied. The first and second vignettes concerned the identification of psychiatric, and non-steroidal anti-inflammatory drugs in patients with mental disorders, namely dementia and bipolar disorder, and the third vignette concerned a cardiovascular drug in a patient with dementia. The third domain focused on barriers in practice and was implemented by asking HCPs the proportion of patients to whom they normally review pharmacotherapeutic records; and by listing potential barriers to PIMs' management in clinical practice, to be ranked on a 5-point agreement Likert scale. The first and third domains were assessed using a 5-item Likert scale, ranging from “Strongly disagree,” to “Strongly agree.” In the domain evaluating actual knowledge, for each clinical vignette, there were four questions scored from 0 to 2.5 points, which accounted for a maximum of 10 points.

Face and content validity were established by an expert panel composed of 12 HCPs from different fields of practice (2 physicians, 5 pharmacists, 2 nurses and 3 academics/researchers). The draft questionnaire was modified following the comments made by the experts. The final version of the questionnaire is available as Supplementary Material S1.

### Data Collection

Data were collected using two different approaches: the first one included the dissemination of the e-questionnaire through the social media, which included Facebook, LinkedIn, and Twitter; the second included the dissemination of the questionnaire through a list of e-mails from our research team. In the first approach, all researchers from our team shared the link in their personal pages, and in private and open social media groups addressing the selected HCPs (e.g., wenurses, wepharmacists, wedoctors). These publications were shared every 2 weeks, and we asked all participants to share the link in their personal pages. In the second approach, the questionnaire was sent via email to a list of professional contacts from the research team. We invited all colleagues that were eligible for the study and asked them to forward the link to other HCPs fitting the inclusion criteria. Reminders were made every 2 weeks in both approaches.

### Data Analysis

Descriptive statistics were used, where numerical variables were expressed using central tendency and dispersion measures and categorical variables as absolute and relative frequencies. The Kolmogorov-Smirnov was used to test the sample distribution. Regarding our main outcome (real knowledge), the median was considered to set the threshold between positive and negative scores. For the total score the threshold was set at 13.75 out of 30 points, and for each individual vignette the threshold was 4 out of 10 points. Therefore, knowledge was divided in three categories (total score over 30): “poor knowledge” (<13.75 points); “intermediate knowledge” ([13.75–20[points; and “advanced knowledge” (≥21 points). Pearson correlation coefficient was calculated to evaluate the strength of correlation between perceived and real knowledge (considering normal distribution of both variables). A value of p <0.05 was considered for Chi-squared test (when comparing categorical variables) and ANOVA test (when comparing numerical variables). Both tests were used to compare perceived and real knowledge and barriers identified between the different professions. IBM SPSS v.21 was used to run the statistical analysis.

### Sensitivity Analysis

Since the questionnaire was disseminated to different countries, we were expecting that differences may be seen, especially if one of the countries had more representativeness comparing to others. Therefore, we performed a sensitivity analysis excluding the responses from other countries to see if there were differences, when analyzing all countries vs. only Portugal.

## Results

### Participants' Characteristics

By the end of the study period, we had 180 HCPs' responses. From those, 3 did not qualify because HCPs did not agree to participate, 5 questionnaires were left blank, and 7 were working exclusively as researcher/lecturer. Thus, our final sample consisted of 165 questionnaires. HCPs were mainly females (*n* = 114; 69.1%), with a mean age of 35.3±11.3 years old {21; 76}. They were practicing in 26 different countries.

From the 165 HCPs that completed the questionnaire, 71.5% (*n* = 118) were pharmacists, 21.2% (*n* = 35) were physicians, and 7.3% (*n* = 12) were nurses. Most HCPs were practicing in the outpatient setting (*n* = 95; 57.6%), followed by inpatient setting (*n* = 70; 42.4%). Sixty-six percent (*n* = 109) had <10 years of experience, and 64.6% (*n* = 106) had a master's degree. Full details are available in Table 1.

**Table 1:** Sociodemographic characteristics.

**Characteristics**	**Total of questionnaires** **(*n* = 165)**
**Age, Mean** **±SD (years)**	35.3 ± 11.3
**Gender**, ***n*** **(%)**	
Male	51 (30.9)
Female	114 (69.1)
**Occupation**, ***n*** **(%)**	
Pharmacist	118 (71.5)
Physician	35 (21.1)
Nurse	12 (7.3)
**Setting where HCPs do their practice**, ***n*** **(%)**		
Ambulatory	95 (57.6)
Hospital	70 (42.4)
**Years of practice**, ***n*** **(%)**	
<5 years	57 (34.5)
5–10 years	52 (31.5)
11–15 years	10 (6.1)
16–20 years	21 (12.7)
More than 20 years	25 (15.2)
**Degree**, ***n*** **(%)**	
Bachelor	28 (17.1)
Integrated Master	62 (37.8)
Master	44 (26.8)
PhD	30 (18.3)
Missing values: 1	

### Knowledge of Different HCPs About Medication Complexities: Perceived vs. Real Knowledge

One-hundred and thirty (76.0%) HCPs confirmed having the knowledge to identify and evaluate the use of PIMs in the elderly in their daily practice, and 70.9% (*n* = 122) considered their knowledge was enough to perform a medication review in elder patients. There were no differences between the different professional classes regarding statement 1 and 2 (*p* = 0.115 and *p* = 0.057, respectively) (Table 2). When exploring differences between the different professional classes on the type of tool used to optimize the pharmacotherapy in older adults, including for PIMs' management, pharmacists seem to more commonly use START/STOPP or Beers criteria when compared to the other two HCPs classes (*p* = 0.023). Conversely, physicians normally use Up-to-date, Medscape, Dynamed, and BMJ-Best Practice (*p* = 0.030).

**Table 2:** Knowledge assessment and practice of physicians, pharmacists, and nurses regarding PIMs management.

**Domain**	**Total sample** **(*n* = 165)**	**Physicians** **(*n* = 35)**	**Pharmacists** **(*n* = 118)**	**Nurses** **(*n* = 12)**	p^*^
**Knowledge**					
Statement 1 – I have knowledge to identify and evaluate the use of PIMs in the elderly in my daily practice, % (*n*)	75.6 (124)	80.0 (28)	77.0 (90)	50.0 (6)	0.115
Statement 2 – I think my knowledge is enough to perform a medication review of my elder patients' therapy, including the use of PIMs, % (*n*)	71 (117)	77.2 (27)	72.1 (85)	41.7 (5)	0.057
Advanced knowledge, % (*n*)	15.4 (25)	14.3 (5)	16.5 (19)	8.3 (1)	0.987
Mean score in the clinical cases, mean ± SD	13.17 ± 7.70	14.29 ± 5.50	13.16 ± 8.08	10.10 ± 9.07	0.269
Mean score in the vignette 1, mean ± SD	4.59 ± 4.08	6.07 ± 3.75	4.32 ± 4.05	2.92 ± 4.37	0.135
Mean score in the vignette 2, mean ± SD	4.86 ± 2.97	4.64 ± 2.30	5.06 ± 3.17	3.44 ± 2.39	0.543
Mean score in the vignette 3, mean ± SD	3.73 ± 3.59	3.57 ± 3.80	3.77 ± 3.49	3.75 ± 4.20	0.959
***Practice***					
Therapeutic regimen revised in <2 out of 10 patients, % (*n*)	56.7 (93)	25.0 (9)	67.5 (77)	41.7 (5)	<0.001^*^
Lack of a specific curricular unit on gerontology in their bachelor/master's degree, % (*n*)	72.8 (118)	77.1 (27)	72.2 (83)	66.7 (8)	0.601
Limited time of appointments/counseling	82.1 (133)	77.2 (27)	83.4 (96)	83.3 (10)	0.884
Scarce clinical tools adjusted to clinical practice	62.6 (102)	60.0 (11)	64.6 (75)	50.0 (6)	0.123

* *p < 0.05*.

**Figure 1:**
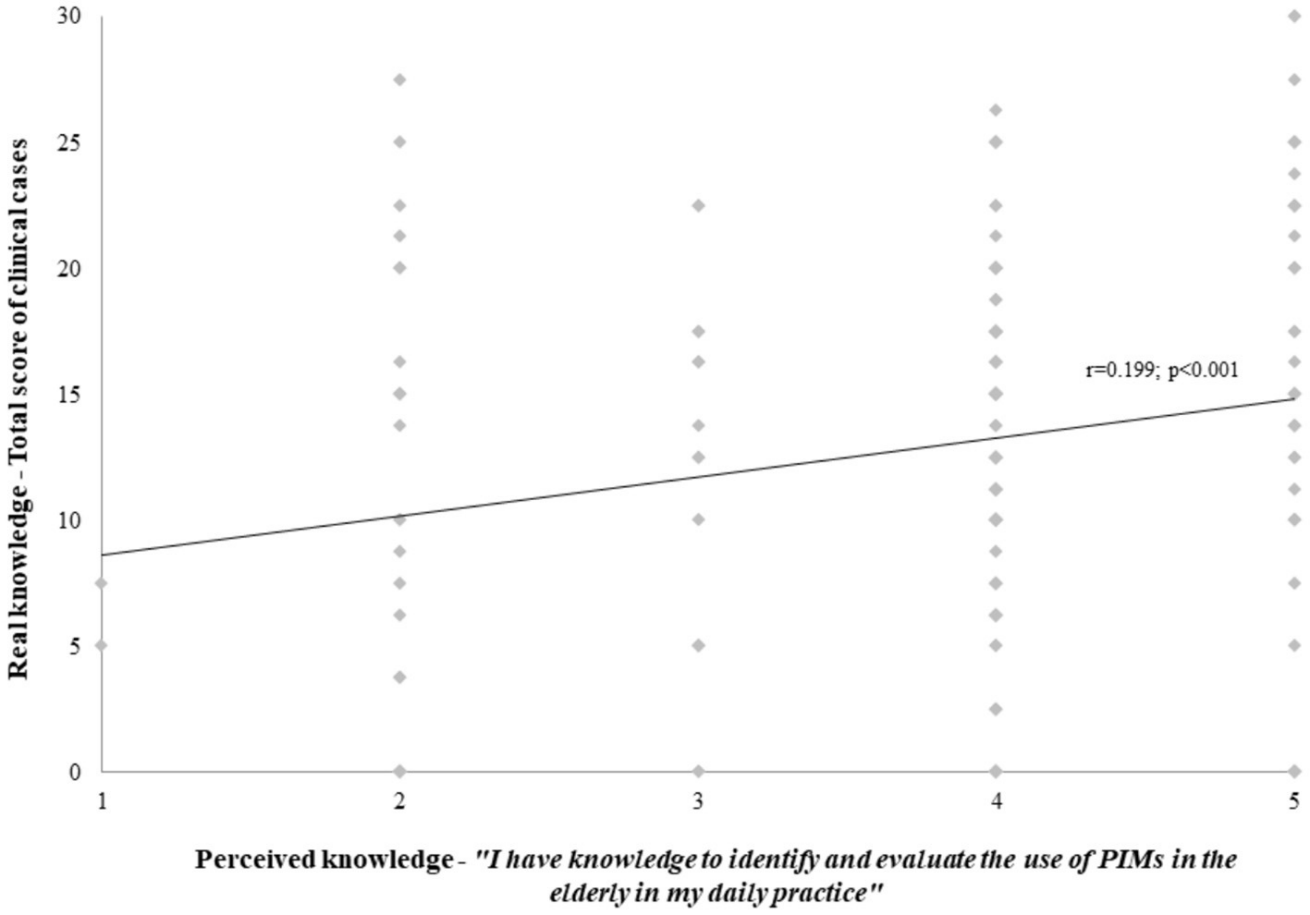
Correlation between real and perceived knowledge.

Concerning actual knowledge, only 15.4% (*n* = 25) of HCPs were classified as having advanced knowledge, but no statistically significant differences were found between different professions (*p* = 0.987). Overall, the mean score of the three clinical vignettes was 13.04 ± 7.69 points {0.0; 30.0}. Pharmacists scored a mean of 13.16 ± 8.08 points, physicians scored 14.29 ± 5.50 points, and nurses scored 10.10 ± 9.07 points, but no differences were found between them (*p* = 0.269) (Table 2). There was a weak correlation between perceived and real knowledge, even though statistically significant (r = 0.199; *p* < 0.001) (Figure 1). Similar results were obtained when analyzing the different professions (Pharmacists – r = 0.205; *p* = 0.027; Physicians – r = 0.025; *p* = 0.887; and Nurses – r = 0.118; *p* = 0.714).

Regarding the clinical vignettes concerning older individuals with dementia and bipolar disorders, the mean score was 4.59 ± 4.08 and 4.86 ± 2.97 points, respectively. Most HCPs were classified as having an intermediate knowledge (*n* = 100; 60.6%) about medication complexities in the elderly with mental disorders.

### Potential Barriers to Medication Complexities' Management in Clinical Practice

Fifty-seven percent (n = 93) of HCPs normally review the pharmacotherapeutic regimen in <2 out of 10 patients in their clinical practice. Differences were found in the average number of patients where the pharmacotherapeutic regimen is reviewed, where physicians tend to review the medication in at least 6 out of 10 patients compared to pharmacists and nurses (*p* < 0.001) (Table 2). Participants agreed that limited time of appointments (81.6%; *n* = 138), lack of a specific curricular unit of gerontology in their bachelor/master degree (72.2%; *n* = 122), and scarce clinical tools adjusted to clinical practice (61.8%; *n* = 105) were the major potential barriers to PIMs' management in clinical practice. There were no differences between barriers perceived by the different HCPs (Table 2).

Other barriers, including lack of interprofessional collaboration, limited access to clinical and laboratory information, no remuneration, fear of deprescribing drugs, and lack of confidence in their own recommendations, were also listed as barriers to clinical management of PIMs.

### Sensitivity Analysis

There were no differences in the results when considering only Portugal in the analysis.

## Discussion

### Main Findings

In this study, we found that most participants felt confident in managing PIMs and no differences were found between physicians, pharmacists, and nurses. However, when evaluated by clinical vignettes, only 15.4% (*n* = 25) of HCPs were considered to have advanced knowledge and no statistical differences were found between different professions. There was a weak correlation between perceived and real knowledge, even though statistically significant. When looking to the clinical vignettes of patients with mental health disorders, we found that even though participants felt confident in managing their therapeutic complexities, only a minority obtained a good score in the knowledge assessment test. Moreover, HCPs agreed that limited time for appointments, lack of a specific curricular unit of gerontology in their bachelor/master's degree, and scarce clinical tools adjusted to clinical practice were major potential barriers to PIMs' management in clinical practice.

In this study, most participants felt confident that their knowledge was enough to perform a medication review in their older patients, which is in accordance with previous studies. Ramaswamy et al. (20) assessed the knowledge, confidence, and barriers to appropriate prescribing in the elderly among family and internal medicine residents and attending doctors in three teaching hospitals in the US and found that 75% felt confident about their prescribing patterns (20). Another study, conducted by Akkawi and Nik Mohamed (18), found a lower degree of confidence (34%) in the ability to recommend appropriate medications for elderly patients; however, no differences were found between physicians and clinical pharmacists (18). These results corroborate our findings. When asked how often HCPs use specific tools that may help in identifying and managing PIMs, most respondents stated to hardly use them (between 0.0 and 20.0%). Other studies have shown similar results, despite HCPs being aware of the existence of such tools (17, 18). This suggests that only a small proportion of HCPs may have heard of explicit tools, and an even smaller proportion may have used it. According to our results, two potential barriers were identified as possible determinants: lack a specific curricular unit of gerontology in pre-graduated studies, and limited availability of clinical tools adjusted to clinical practice. This means that probably we can have two scenarios: the first one, where they never heard of explicit tools, because they did not have a specific curricular unit of gerontology or appropriate pharmacotherapy for the elderly; and the second one, where they heard of explicit tools and they know where to find them, but they think that these tools are not adapted to clinical practice as they are mostly available as extensive tables. This last finding associated to the fact that most participants agreed that limited time for appointments is a relevant constraint for PIMs' management in clinical practice, seems to corroborate out second hypothesis.

Pharmacists were the professional class that reported more frequent use of explicit criteria, compared to physicians, and nurses. This may be linked to the fact that some curricular programmes of the pharmacy degree have changed in recent years, including topics on medicines management in the elderly, including medication review. However, there was no statistically significant differences between the scores obtained by pharmacists and the other two professional classes.

When evaluating actual knowledge using clinical vignettes, we observed that only a small proportion of the sample was classified as having advanced knowledge, in accordance with previous studies (17, 18, 20). In our study physicians showed a better knowledge in the first vignette focusing on psychiatric drugs in elderly patients with cardiovascular disease, whereas pharmacists showed a better score in the second vignette focusing on NSAIDs and benzodiazepines in patients with osteoarticular disease. This could mean that pharmacists are more aware of the potential adverse drug events (ADEs) of these drugs, whereas physicians are more used to deal with certain medications that may increase the cardiovascular risk; however, some of these potential ADEs may be found in other platforms like Up-to-date or Medscape, which were the most frequently used tools by physicians. The use of benzodiazepines and the risk of falls and the risk of gastrointestinal bleeding associated with the use of NSAIDs are associations very well established, particularly for the elderly population. However, there was a weak correlation between perceived and real knowledge, including between the different professions. This may suggest that in the future, changes on how future HCPs are evaluated should be adapted to a more real-world situation using case studies, instead of theoretical examination only. This has been defended by Miller since the 90's, and later adapted by various researchers and professional organizations focused on competency training and continuous professional development, to highlight the difference between knowledge and competency, knowing how and being able to competently deliver (21).

### Impact on Practice

To our best knowledge, this is one of the few studies assessing knowledge and practice of different HCPs (physicians, pharmacists, and nurses) on pharmacotherapy optimization for older adults with mental health disorders, including PIMs' management. Considering global aging, it is imperative for HCPs to be prepared to manage multimorbidity and polypharmacy as new challenges in clinical practice. PIMs are one example of another challenge that HCPs are very likely to face in practice and demand specific knowledge and skills. Therefore, future work will focus on the inclusion of these extensive lists in digital clinical-decision support systems to more efficiently help HCPs to manage medication in the elderly, and also to foster more intense interprofessional collaboration where all contribute along the patient pathway, avoiding silos and information mismatches.

### Limitations

This study has some limitations worth acknowledging, including the inability to estimate a sample size given the absence of data on physicians, pharmacists, and nurses accessing social media worldwide. It is also important to mention that the study period (July-October 2019) may have influenced our sample size, since most of those months are coincident with summer holidays, where HCPs are less available to participate in research studies. Additionally, this sample may represent a self-selected sample, as many of the participants seem to be HCPs involved in the Geriatric field and, therefore, their knowledge may be higher when compared to others less involved. There is also a disproportionality in terms of the number of different HCPs included, i.e., pharmacist represents most of the sample (72.0%), which may influence the results. There are also no differences between different medical specialties, which may be justified by the self-selected sample in which most of the physicians that agreed to participate, may have a higher knowledge on geriatrics or even a subspeciality in this field. It is also important to acknowledge that the different undergraduate curriculum of the different healthcare professions may influence their knowledge in this field. Clinical vignettes were based on the Beers criteria, but the assessment of clinical knowledge considered may be questioned. However, we do believe that this approach is a better way than using implicit criteria that lies more on the clinical judgement and hence in previous knowledge of pharmacotherapy. The fact that most of the criteria presented on those lists is related to ADRs known to be commonly experienced by the elderly (e.g., NSAIDs should be avoided due to an increased risk of bleeding) was considered a proof of validity *per se*. A more qualitative approach to access the potential barriers to PIMs could have been considered and would eventually result in more in-depth material for future work. Finally, our results cannot be generalized given the limited sample.

### Conclusions

Most of the HCPs felt confident to manage medication complexities in elder patients with mental disorders, but only a minority obtained a good score in the knowledge assessment test. There were no differences between physicians, pharmacists, and nurses concerning their confidence and knowledge about optimizing the pharmacotherapy in older adults, including PIMs management. Additionally, a weak correlation between perceived and real knowledge was found. Main barriers identified included structural barriers (tools unfit for practice) and process barriers (time), suggesting education *per se* will not necessarily lead to optimized pharmacotherapy in the elderly.

## Data Availability Statement

The raw data supporting the conclusions of this article will be made available by the authors, without undue reservation.

## Ethics Statement

The studies involving human participants were reviewed and approved by Ethics Committee of the Faculty of Pharmacy, University of Lisbon. The patients/participants provided their written informed consent to participate in this study.

## Author Contributions

FAC, HL, and JA conceived and designed the study. JA and JGM collected, analyzed, and interpreted the data. JA prepared the manuscript. All the authors have critically reviewed the manuscript until its final version. All authors contributed to the article and approved the submitted version.

## Funding

This work is financed by national funds through the FCT - Foundation for Science and Technology, I.P., under the project UIDB/04585/2020.

## Conflict of Interest

The authors declare that the research was conducted in the absence of any commercial or financial relationships that could be construed as a potential conflict of interest.

## Publisher's Note

All claims expressed in this article are solely those of the authors and do not necessarily represent those of their affiliated organizations, or those of the publisher, the editors and the reviewers. Any product that may be evaluated in this article, or claim that may be made by its manufacturer, is not guaranteed or endorsed by the publisher.

## References

[1] Brahma D Wahlang J Marak M. Ch Sangma M. Adverse drug reactions in the elderly. J Pharmacol Pharmacother. (2013) 4:91. 10.4103/0976-500X.110872 23761706 PMC3669588

[2] Suzman R Beard J. Global health and aging. NIH Publ. (2011) 1:273–7.

[3] Minuti A Patrone V Giuberti G Spigno G Pietri A Battilani P. Nutrition and ageing. Stud Health Technol Inform. (2014) 203:112–21. 26630518

[4] Nobili A Garattini S Mannucci P. Multiple diseases and polypharmacy in the elderly: challenges for the internist of the third millennium. J Comorb. (2011) 1:28–44. 10.15256/joc.2011.1.4 29090134 PMC5556419

[5] Davies EA O'Mahony MS. Adverse drug reactions in special populations - the elderly. Br J Clin Pharmacol. (2015) 80:796–807. 10.1111/bcp.12596 25619317 PMC4594722

[6] Masnoon N Shakib S Kalisch-Ellett L Caughey GE. What is polypharmacy? A systematic review of definitions. BMC Geriatr. (2017) 17:230. 10.1186/s12877-017-0621-2 29017448 PMC5635569

[7] Alhawassi TM Krass I Bajorek B Pont LG. A systematic review of the prevalence and risk factors for adverse drug reactions in the elderly in the acute care setting. Clin Interv Aging. (2014) 9:2079–86. 10.2147/CIA.S71178 25489239 PMC4257024

[8] Schmiedl S Rottenkolber M Szymanski J Drewelow B Siegmund W Hippius M. Preventable ADRs leading to hospitalization — results of a long-term prospective safety study with 6,427 ADR cases focusing on elderly patients. Expert Opin Drug Saf. (2018) 17:125–37. 10.1080/14740338.2018.1415322 29258401

[9] Nyborg G Brekke M Straand J Gjelstad S Romøren M. Potentially inappropriate medication use in nursing homes: an observational study using the NORGEP-NH criteria. BMC Geriatr. (2017) 17:1–11. 10.1186/s12877-017-0608-z 28927372 PMC5606129

[10] Mahlknecht A Krisch L Nestler N Bauer U Letz N Zenz D. Impact of training and structured medication review on medication appropriateness and patient-related outcomes in nursing homes: Results from the interventional study InTherAKT. BMC Geriatr. (2019) 19:257. 10.1186/s12877-019-1263-3 31533630 PMC6749664

[11] Roughead EE Vitry AI Caughey GE Gilbert AL. Multimorbidity, care complexity and prescribing for the elderly. Aging Health. (2011) 7:695–705. 10.2217/ahe.11.64

[12] Shrivastava A de Sousa A Lodha P. Polypharmacy: a challenge for community psychiatrists. Psychiatr Times. (2019) 7:25–35.

[13] Fornaro M de Berardis D Koshy AS Perna G Valchera A Vancampfort D. Prevalence and clinical features associated with bipolar disorder polypharmacy: a systematic review. Neuropsychiatr Dis Treat. (2016) 12:719–35. 10.2147/NDT.S100846 27099503 PMC4820218

[14] Fornaro M Solmi M Stubbs B Veronese N Monaco F Novello S. Prevalence and correlates of major depressive disorder, bipolar disorder and schizophrenia among nursing home residents without dementia: Systematic review and meta-analysis. Br J Psychiatry. (2020). 216: 6–15. 10.1192/bjp.2019.5 30864533

[15] Vaismoradi M Jamshed S Lorenzl S Paal P. Prn medicines management for older people with long-term mental health disorders in home care. Risk Manag Healthc Policy. (2021) 14:2841–9. 10.2147/RMHP.S316744 34262371 PMC8274703

[16] von Elm E Altman DG Egger M Pocock SJ Gøtzsche PC Vandenbroucke JP. The Strengthening the Reporting of Observational Studies in Epidemiology (STROBE) Statement: Guidelines for Reporting Observational Studies. Available online at: http://www.epidem.com/ (accessed April 16, 2022).

[17] Foong RTK Sellappans R Loo JSE. Awareness of beers criteria and knowledge of potentially inappropriate medications among community pharmacists in the Klang Valley, Malaysia. J Eval Clin Pract. (2020) 26:165–71. 10.1111/jep.13180 31168913

[18] Akkawi ME Nik Mohamed MH. Are physicians and clinical pharmacists aware and knowledgeable enough about inappropriate prescribing for elderly patients? Findings from Malaysia. Eur J Hosp Pharm. (2018) 25:E29–34. 10.1136/ejhpharm-2017-001391 31157063 PMC6457149

[19] Fadare JO Obimakinde AM Enwere OO Desalu OO Ibidapo RO. Physician's knowledge of appropriate prescribing for the elderly—a survey among family and internal medicine physicians in Nigeria. Front Pharmacol. (2019) 10:1–8. 10.3389/fphar.2019.00592 31214031 PMC6554676

[20] Ramaswamy R Maio V Diamond JJ Talati AR Hartmann CW Arenson C. Potentially inappropriate prescribing in elderly: Assessing doctor knowledge, confidence and barriers. J Eval Clin Pract. (2011) 17:1153–9. 10.1111/j.1365-2753.2010.01494.x 20630004

[21] Miller GE. The assessment of clinical skills/competence/performance. Acad Med. (1990) 65(9 Suppl):S63–S67. 10.1097/00001888-199009000-00045 2400509

