# Supplementary material for: Healthcare Professionals' Views on the Management of Medication Complexities in the Elderly With Mental Health Disorders: A Cross-Sectional Study
Source: Front Psychiatry. 2022 May 23;13:885216. doi: 10.3389/fpsyt.2022.885216 (PMC9168079; doi:10.3389/fpsyt.2022.885216)
Supplement: Supplementary file 1 [file Table_1.DOCX]

**Supplementary Material S1 – Questionnaire**

**Section 1 – Sociodemographic data**

1 – Age

2 – Country

3 – Sex:

Male

Female

4 – Occupation:

Physician

Pharmacist

Nurse

Other. Please state which:

4.1. – If you selected “Physician”, please indicate which specialty you have (or area you work in)___________

4.2. – If you selected “Pharmacist”, please indicate which specialty you have (or area you work in)___________

4.3. – If you selected “Nurse”, please indicate which specialty you have (or area you work in)___________

5 – For how many years have you been working?

Less than 5 years

5 to 10 years

11-15 years

16-20 years

More than 20

I am not currently working

6 – Academic degree

Bachelor

Integrated Master

Second Cycle Master

PhD

**Section 2 – Perceived knowledge**

Please consider the following definition of medication review (adapted from Griese-Mammen, 2018): is a structured evaluation of patient’s medication with the aim of optimizing medicines use and improving health outcomes. This entails detecting PIMs or contraindicated medications and recommending or implementing interventions.

1 – Considering a PIM as a medicine where the risk of adverse drug reactions (ADRs) overweights the clinical benefit and for which there is a safer alternative available, I have the knowledge to identify and evaluate the use of PIMs in the elderly in my daily practice

Strongly disagree

Partially disagree

Do not disagree/do not agree

Partially agree

Strongly agree

2 – I think my knowledge is enough to perform a medication review of my elder patients’ therapy, including the use of PIMs.

Strongly disagree

Partially disagree

Do not disagree/do not agree

Partially agree

Strongly agree

3 – How often do you use clinical decision support systems to perform medication review in your elder patients?

Never (0-20%)

Rarely (20-40%)

Sometimes (40-60%)

Most of the times (60-80%)

Always (80-100%)

4 – How often do you use each of the following tools?

Beers Criteria

Never (0-20%)

Rarely (20-40%)

Sometimes (40-60%)

Most of the times (60-80%)

Always (80-100%)

START/STOPP criteria

Never (0-20%)

Rarely (20-40%)

Sometimes (40-60%)

Most of the times (60-80%)

Always (80-100%)

PRISCUS List

Never (0-20%)

Rarely (20-40%)

Sometimes (40-60%)

Most of the times (60-80%)

Always (80-100%)

Up to date

Never (0-20%)

Rarely (20-40%)

Sometimes (40-60%)

Most of the times (60-80%)

Always (80-100%)

Medscape

Never (0-20%)

Rarely (20-40%)

Sometimes (40-60%)

Most of the times (60-80%)

Always (80-100%)

Dynamed

Never (0-20%)

Rarely (20-40%)

Sometimes (40-60%)

Most of the times (60-80%)

Always (80-100%)

BMJ – Best Practice

Never (0-20%)

Rarely (20-40%)

Sometimes (40-60%)

Most of the times (60-80%)

Always (80-100%)

**Section 3 – Real Knowledge**

In this section you can find three clinical cases, where you should answer according to what you would do in your clinical practice.

1 – Ms. DS, 77 years old, with hypertension, dyslipidaemia, type 2 diabetes, and dementia, has been prescribed with atorvastatin 20 mg (once daily), perindopril 4 mg (once daily), sitagliptin 100 mg (once daily), and olanzapine 5 mg (once daily).

1.1. Among the medication list, do you identify any PIM?

Yes

No

1.2. Which medicine(s) would you classify as PIM?

Atorvastatin

Perindopril

Sitagliptin

Olanzapine

1.3. What do you consider to be the reason why the previously medicine(s) is(are) considered as PIM?

Atorvastatin – risk of muscular pain and, in more severe cases, rhabdomyolysis

Association of olanzapine with sitagliptin – high risk of hyperglycaemia with olanzapine use

Association of perindopril with atorvastatin – risk of renal failure

Olanzapine – increase of cardiovascular risk em patients with previous history of cardiovascular disease

Other reasons. Which?

1.4. What do you think would be the most appropriate action in this case?

Inform the treating physician/prescriber of the need to deprescribe the medication(s) and suggest a safer alternative (in case of being the prescriber, I would deprescribe the medicine myself)

Suggest dose adjustment of the medicine(s)

Suggest keeping the medicine(s), because there is no effective alternative available, even though is not safe

There is no need to make any suggestion

Other actions. Which?

2. Mr. MC, 79 years old, with bipolar disorder and musculoskeletal disease, has been prescribed with lithium 200 mg (once daily), alprazolam 1 mg (thrice daily), paracetamol 1000mg (thrice daily), and naproxen 500mg (twice daily when needed).

2.1. Among the medication list, do you identify any PIM?

Yes

No

2.2. Which medicine(s) would you classify as PIM?

Lithium

Alprazolam

Paracetamol

Naproxen

2.3. What do you consider to be the reason why the previous medicine(s) is(are) considered as PIM?

Lithium – risk of toxicity in patients with renal failure

Alprazolam – high risk of falls in the elderly

Paracetamol – contraindicated in patients with heart failure

Naproxen – risk of GI bleeding in patients aged 75 or older

Other reasons. Which?

2.4. What do you think would be the most appropriate action in this case?

Inform the physician/prescriber of the need to deprescribed the medication and suggest a safer alternative (in case of being the prescriber, I would deprescribed the medicine myself)

Suggest dose adjustment of the medicine

Suggest keeping the medicine, because there is no effective alternative available, even though is not safe

There is no need to make any suggestion

Other actions. Which?

3. Mr. HN, 88 years old, with dyslipidaemia, heart failure, previous history of myocardial infarction, and dementia, has been prescribed with rosuvastatin 10 mg (once daily), enalapril 5 mg (once daily), furosemide 40 mg (once daily), bisoprolol 5 mg (once daily), digoxin 0.25 mg (once daily), and galantamine 8 mg (once daily). This patient does not have renal failure.

3.1. Among the medication list, do you identify any PIM?

Yes

No

3.2. Which medicine(s) would you classify as PIM?

Galantamine

Rosuvastatin

Bisoprolol

Digoxin

There are no PIMs

3.3. What do you consider to be the reason why the previous medicine(s) is(are) considered as PIM?

Association of rosuvastatin with digoxin – increased risk of rhabdomyolysis

Digoxin – in doses ≥ 0.125mg can cause toxicity in older patients with heart failure

Association of digoxin with bisoprolol – increased risk of hyperkalaemia

Association of galantamine with bisoprolol – increased risk of bradycardia

Other reasons. Which?

3.4. What do you think would be the most appropriate action in this case?

Inform the physician/prescriber of the need to deprescribed the medication and suggest a safer alternative (in case of being the prescriber, I would deprescribed the medicine myself)

Suggest dose adjustment of the medicine

Suggest keeping the medicine, because there is no effective alternative available, even though is not safe

There is no need to make any suggestion

Other actions. Which?

**Section 4 - Structural or procedural barriers in clinical practice**

1. Considering the last 10 patients you saw, in how many of them, did you undertake a medication review?

Less than 2 patients

3 to 4 patients

5 to 6 patients

7 to 8 patients

9 to 10 patients

2. Please select your level of agreement on the barriers around the knowledge of PIMs

Lack of a specific curricular unit on gerontology in your bachelor/master degree

Strongly disagree

Partially disagree

Do not disagree/do not agree

Partially agree

Strongly agree

Lack of clinical tools adjusted to clinical practice

Strongly disagree

Partially disagree

Do not disagree/do not agree

Partially agree

Strongly agree

Lack of time available for each patient

Strongly disagree

Partially disagree

Do not disagree/do not agree

Partially agree

Strongly agree

Please list any other barriers you consider relevant.
